# Role of MSC‐derived small extracellular vesicles in tissue repair and regeneration

**DOI:** 10.3389/fcell.2022.1047094

**Published:** 2023-03-01

**Authors:** Bruna Andrade Aguiar Koga, Letícia Alves Fernandes, Paula Fratini, Mari Cleide Sogayar, Ana Claudia Oliveira Carreira

**Affiliations:** ^1^ Cell and Molecular Therapy Group (NUCEL), School of Medicine, University of São Paulo, São Paulo, Brazil; ^2^ Department of Surgery, Faculty of Veterinary Medicine and Animal Science, University of São Paulo, São Paulo, Brazil; ^3^ Biochemistry Department, Chemistry Institute, University of São Paulo, São Paulo, Brazil; ^4^ Center for Natural and Human Sciences, Federal University of ABC, São Paulo, Brazil

**Keywords:** extracellular microvesicles, exosomes, tissue regeneration, regenerative medicine, immunomodulation

## Abstract

Mesenchymal stem cells (MSCs) are crucial for tissue homeostasis and repair, secreting vesicles to the extracellular environment. Isolated exosomes were shown to affect angiogenesis, immunomodulation and tissue regeneration. Numerous efforts have been dedicated to describe the mechanism of action of these extracellular vesicles (EVs) and guarantee their safety, since the final aim is their therapeutic application in the clinic. The major advantage of applying MSC-derived EVs is their low or inexistent immunogenicity, prompting their use as drug delivery or therapeutic agents, as well as wound healing, different cancer types, and inflammatory processes in the neurological and cardiovascular systems. MSC-derived EVs display no vascular obstruction effects or apparent adverse effects. Their nano-size ensures their passage through the blood–brain barrier, demonstrating no cytotoxic or immunogenic effects. Several *in vitro* tests have been conducted with EVs obtained from different sources to understand their biology, molecular content, signaling pathways, and mechanisms of action. Application of EVs to human therapies has recently become a reality, with clinical trials being conducted to treat Alzheimer’s disease, retina degeneration, and COVID-19 patients. Herein, we describe and compare the different extracellular vesicles isolation methods and therapeutic applications regarding the tissue repair and regeneration process, presenting the latest clinical trial reports.

## Graphical Abstract

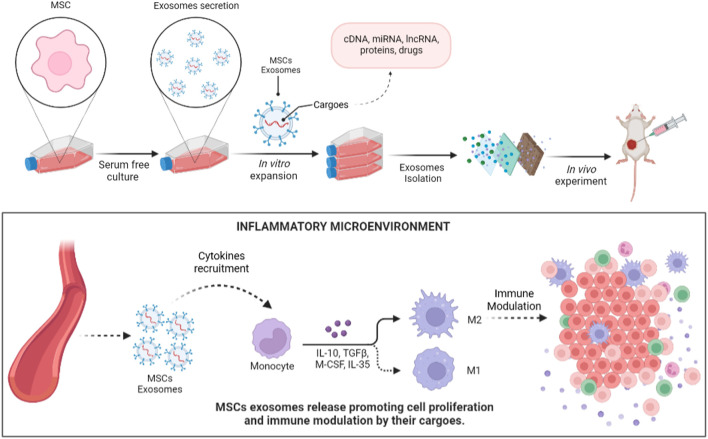

## Background

Regenerative medicine has faced great challenges in the search for alternatives to ensure an effective treatment to accelerate the tissue regeneration process without altering its phases, especially for wound healing, in clinical, morphophysiological, and molecular environments (Lin et al., 2011; Choi et al., 2012; Waycaster et al., 2016). Instead of whole organ/tissue transplantation, a novel approach is cell therapy characterized by the use of cells with immunomodulatory properties as treatment. In order to do that, Mesenchymal/stromal stem cells have been used as an important resource for cell therapy being widely described for several applications, such as the treatment of wounds and ulcers of Diabetes mellitus (Cao et al., 2017; Di et al., 2017) and regeneration of several tissue types (Brett et al., 2017; Thangarajah et al., 2017), as an alternative for cancer treatment, and in studies of cancer biology (Gomes et al., 2017; Yao et al., 2017). Despite the accumulated knowledge about stem cell therapeutic applications, several mechanisms involved in the regenerative process have been unclear for a long time. Currently, some light has been shed on this problem, bringing attention to the extracellular components released by these cells. The discovery of extracellular vesicles (EVs) raised the question of whether these components would be the actual effectors responsible for MSC-mediated cell therapy (Akyurekli et al., 2015).

This recent finding drew the attention of the scientific community with several questions emerging: 1) what are these vesicles released by MSCs?; 2) what are the mechanisms of release of these vesicles?; 3) what are the characteristics of these vesicles and the molecular components of their content; and 4) what are the possible mechanisms of action of these secreted molecules and their therapeutic potential, among several other questions regarding EVs (Pankajakshan and Agrawal, 2014; Marote et al., 2016; Derkus et al., 2017).

EVs were first described in the 1980s as vesicles of endosomal origin, which were observed to be released during the maturation of reticulocytes, as a consequence of multi-vesicular endosome fusion to the plasma membrane (Johnstone et al., 1987). Recent studies report EVs as active participants in biological, regenerative, inflammatory, and pathological processes (De Toro et al., 2015; Wang B et al., 2019). Their molecular content has been the subject of investigation seeking to uncover the basis for their efficacy in several therapies. It is suggested that EVs play a fundamental role in intercellular communication, acting as bioindicators (An et al., 2015) or therapeutic agents, in addition to promoting a better understanding of pre-existent cell therapy (Phinney and Pittenger, 2017). Therefore, this review aims to highlight the most recent studies on EVs derived from different types of MSCs, discussing the different methodological approaches for their isolation and use in tissue regeneration processes.

## Mesenchymal/stromal cells

**FIGURE 1:**
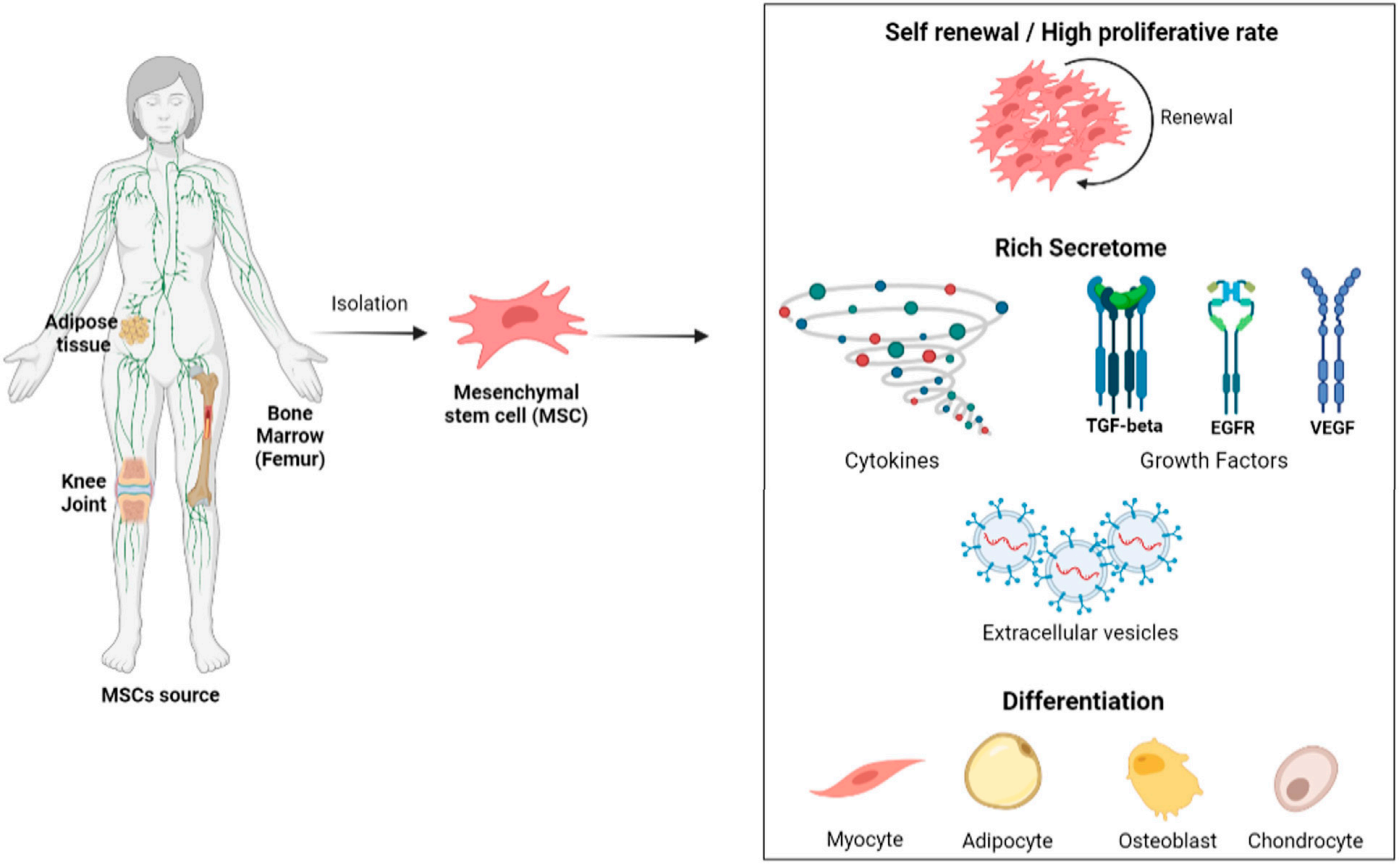
Mesenchymal stem cells (MSCs) could be isolated from adipose tissue, bone marrow, and different joints (especially to obtain chondrocytes). After the isolation method, they should be able to differentiate into adipocytes, osteoblasts, and chondrocytes. Several characteristics draw attention to studies with MSCs, but the most attractive is their self-renewal potential, as well as other types of stem cells, and the presence of a secretome with different molecules such as cytokines, growth factors, and extracellular vesicles. Image created on BioRender.

Mesenchymal/stromal stem cells (MSCs) are multipotent non-hematopoietic stem cells, which are natural residents of adult tissues (Figure 1) and are involved in tissue homeostasis and recovery from injuries (Dominici et al., 2006; Katsuda and Ochiya, 2015). These cells can be obtained from many sources, such as the umbilical cord, peripheral blood, dental tissue, and liver. However, the most common and best characterized have been bone marrow and adipose tissue (Wang et al., 2020). According to Dominici et al. (2006) and the International Society for Stem Cell Therapy (ISSCR), MSCs are characterized by three main criteria: a) plastic adherence; b) expression of CD105, CD73, and CD90 and non-expression of CD45, CD34, CD14, CD19, and HLA class II; and c) differentiation into osteoblasts, chondroblasts, and adipocytes.

Their principal mechanism of action is through immunomodulation of innate and adaptative immune response interacting with T cells, B cells, natural killer (NK) cells, macrophages, and dendritic cells and by paracrine activity *via* secretome (Song N et al., 2021). Also, immune suppression of MSCs is influenced by the balance of their own secretion with the systemic and local environment inflammatory cytokines (Ankrum et al., 2014). Due to their immune tolerance, application of allogeneic or autologous MSCs has been widespread in clinical trials (Samsonraj et al., 2017). Together with the ability of MSCs to differentiate into other cell types *ex vivo*, much evidence is available on their therapeutic potential (Toh et al., 2016).

The ability to secrete soluble factors exerting paracrine activity in the microenvironment exemplifies the beneficial effects of MSCs in tissue repair and regeneration (Abreu et al., 2016). Among the mediators released by their secretome are cytokines, microRNAs (miRNA), growth factors, and EVs (Raposo and Stoorvogel, 2013).

MSCs have already been used in different lung diseases, cardiovascular repair, cancer treatment, immunological disease, spinal cord injury, and bone and cartilage replacement. Recent clinical trial application of these cells can be found in Table 3.

## Extracellular vesicles

**FIGURE 2:**
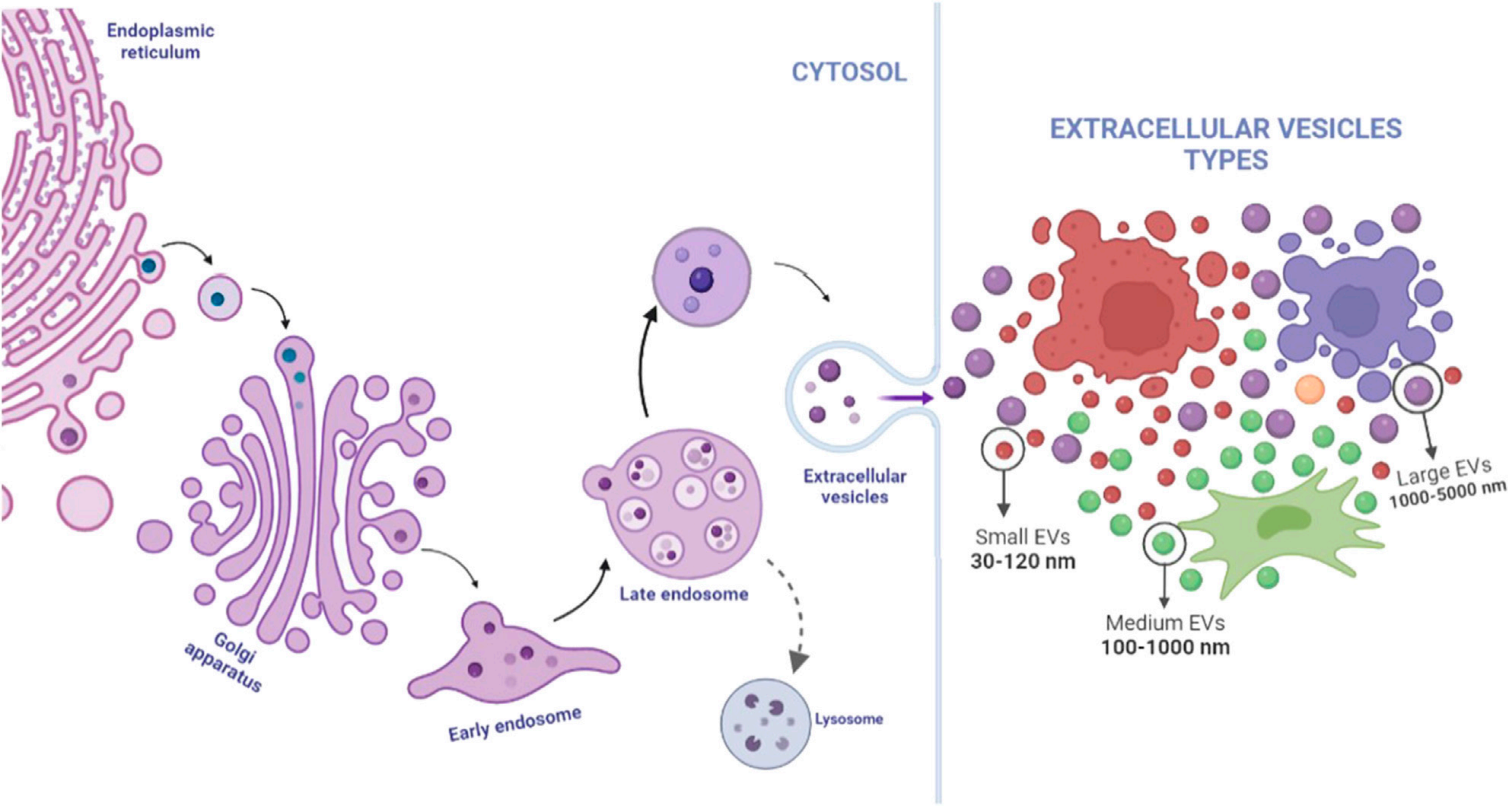
Extracellular vesicles are a generic name for many kinds of vesicles released by cells; their origin and size are determinants in their classification. Exosomes present size between 30 and 120 nm, and an endosome being initially formed in cell cytosol from the early endosomes until late endosome release by the influence of specific proteins such as ESCRT and Rab family and could be excreted by any type of cells, including MSCs, tumor cells, and, even, immune cells. Microvesicles could present size within the range of 100–1,000 nm and could be released by any cells, whereas apoptotic bodies are the biggest group, excreted only for apoptotic cells. Image created on BioRender.

EVs include a broad spectrum of vesicles secreted by various cell types, originating from different body fluids (Table 1). This name is also used as a general term which includes: exosomes, ectosomes, oncosomes, release vesicles, apoptotic bodies, and microvesicles (Figure 2) (Choi and Lee, 2016; Elahi et al., 2019). To date, the International Society of Extracellular Vesicles (ISEV) recommends the application of EVs as an operational term and suggests classifications according to size (small, medium, or large EVs), biochemical composition (CD63^+^ EVs/CD81^+^ EVs) or conditions, and cell origin (apoptotic bodies and hypoxic EVs) (Théry et al., 2018).

**TABLE 1:** Different sources used to isolate EVs, methods of isolation, and effectiveness of each project.

Source of EVs	Application/purpose	Method of EVs isolation	Quantity of EVs used/obtained*	Effectiveness/results	References
Human plasma	Develop a single-step protocol to isolate EVs from plasma aiming at less contamination by proteins and high-density lipoproteins (HDL)	Size-exclusion chromatography (SEC) with Sepharose CL-2B column	1.5 ml of platelet-free plasma was collected in 26 fractions of 0.5 ml. Fractions 9–12 presented the highest concentrations of particles (46%) and 4 μg EV protein (Western blot)	With this method, there is no risk of protein complex formation, being fast and inexpensive, with the recovery of vesicles with Sepharose CL-2B SEC being 43%, compared to 2%–80% by ultracentrifugation. Display good vesicle recovery with almost complete removal of contaminants	Böing et al. (2014)
Visceral adipose tissue	Evaluate how a high-fat diet influences colitis through exosomes derived from visceral adipose tissue. This work also isolated exosomes from the liver, skeletal muscle, and subcutaneous adipose tissue	Ultracentrifugation and ExoQuick Kit	Tissues with less than 1 cm^3^ cultured for 24 h in a serum-free medium. Recovery not specified	A high-fat diet changed miRNA-exosome profile switching from an anti-inflammatory to a pro-inflammatory phenotype, increasing intestinal inflammation *via* M1 macrophage	Wei M et al. (2020)
Human and rat plasma	Compare the efficiency of SEC and ultracentrifugation to isolate exosomes with minimum content of albumin as contaminant	size exclusion chromatography (SEC) *versus* ultracentrifugation (UC)	UC: 7 ml twofold diluted plasma SEC: 0.5 ml of filtered platelet-free plasma was diluted and loaded with 10 ml of bed volume (results presented by Western blot)	SEC is more suitable than UC for the exos isolation from blood without significant albumin contamination, but the efficiency needs to be improved	Baranyai et al. (2015)
Conditioned medium (CM) from mouse neuroblastoma and myoblast cells (N2a and C2C12 lines)	Evaluated a novel liquid chromatography technique for EV purification: using core bead chromatography	Bind-elute (BE) size-exclusion chromatography (SEC)	20 ml of filtered conditioned medium supernatant recovery about 80% with values equal to (N2a) 1.17 × 10^10^ *P*/ml and (C2C12) 1.32 × 10^10^ *P*/ml	BE-SEC columns can purify EVs in a reliable and scalable fashion with yields ranging from 70% to 80%, purity comparable to UC, and it is a fast procedure (85–150 min)	Corso et al. (2017)
CM of breast cancer MCF7 and MDA-MB-231 line	Use ultrafiltration/diafiltration for the purification of exosomes from the CM cell lysate with 3 kDa or 100 kDa Amicon® Pro device Purification + TEI	Ultrafiltration-based method	—	Higher protein concentration and higher relative absorbance units for lipids in the 3-kDa or 10-kDa membrane indicated that this device was a better choice for exosome sample preparation	Gutierrez et al. (2013)
Porcine milk	Evaluated whether porcine milk-derived exosomes could attenuate PLS-induced intestinal epithelial inflammation by downregulating inflammatory and apoptosis pathways *via* miRNAs	Ultracentrifugation	∼50 ml fresh porcine milk	Porcine milk-derived exosomes appear to protect the intestine from LPS-induced injury by decreasing inflammation and apoptosis through the inhibition of the NF-ĸβ and p53 pathways	Xie et al. (2019)
			Exosome recovery was not specified		
Urine EVs	Propose a nanowire-based methodology for collecting urine EV-encapsulated miRNAs that unveils massive numbers of urinary miRNAs of different sequences that potentially serve as biomarkers for cancer not only for urologic but also for non-urologic malignancies	Nanowires anchored into a microfluidic substrate	1 ml urine	The device could achieve higher efficiency for *in situ* extraction of urine EV-encapsulated miRNAs compared to the most popular conventional method of ultra- centrifugation. Could extract a much larger variety of species of miRNAs despite the smaller volume and could find cancer-related miRNAs from urine samples of just 1 ml for not only urologic malignancies but also non-urologic ones	Yasui et al. (2017)
			Collection efficiency (small RNA yield)		
			0.194 ± 0.028 ng/ml		
			Extraction species of urinary miRNAs being identified		
			749, 822, 1,111 (*n* = 3)		
Human blood serum	Evaluation of six different kit isolation methods	ExoQuick, ExoEasy, Exospin, ME kit, ExoQuick Plus, ExoFlow	The serum volume follows the manufacturer’s instructions but varies from 250 μL to 4,000 μl. The exosome concentration, also, varies: 44 × 10^13^ P/L; 18 × 10^13^ P/L; 64 × 10^13^ P/L; 0,13 × 10^13^ P/L; 59 × 10^13^ P/L; 10 × 10^13^ P/L (values ordered as in column of Methods)	Regarding particle counts, Exo-spin, exoEasy, and ExoQuick isolated similar values, on the contrary, exoEasy and ExoQuick Plus presented the highest purity levels. An important observation raised was that depending on the KIT chosen, the molecules and their respective concentration could vary	Macías et al. (2019)
Human-breast milk	Analyze the potential protective effect of human-breast milk exosomes from oxidative stress in intestinal epithelial cells	Ultracentrifugation	Initial volume not specified. Recovery of 3–9 × 10^8^ particles/ml	Breast milk exosomes seem to protect against cell toxicity induced by H_2_O_2_ with their own cargo. Also, human milk exosomes could be used in neonates with intestine injury	Martin et al. (2018)
M2-macrophage	Investigate the roles of M2-macrophage-derived exosomes in vascular smooth muscle cell (VSMC) differentiation, including the mechanisms after stent implantation	Ultracentrifugation	Not specified	M2-macrophage-derived exosomes promote re-endothelization and VSMC differentiation *via* the MAPK (AP-1) pathway	Yan et al. (2020)
Wistar rats’ blood plasma	Investigate the effectiveness of UC and SEC methods of exosome isolation	Ultracentrifugation (UC) and Size Exclusion Chromatography (SEC) with Sepharose CL-4B, Sepharose 2B, and Sephacryl S-400	Ultracentrifugation	SEC is more suitable than UC, especially due their low albumin contamination. However, SEC efficiency should be improved	Baranyai et al. (2015)
			7 ml of plasma		
			Recovery not specified		
			Size exclusion chromatography (SEC)		
			0.5 ml of filtered plasma		
			Recovery not specified		
Human blood plasma and cell culture medium	Characterize and compare extracellular vesicles isolated from plasma and cell culture medium using SEC, polyethylene glycol (PEG), and protein organic solvent precipitation (PROSPR)	SEC PEG PROSPR	Size exclusion chromatography (SEC)	Comparing all three methods, the SEC protocol was the most efficient and most capable of removing most protein contamination and maintaining the vesicular structure and conformation of extracellular vesicles	Gámez-Valero et al. (2016)
			2 ml of filtered plasma		
			Recovery not specified		
			Polyethylene glycol (PEG)		
			2 ml of plasma in 50% PEG6000 (final concentration: 10%)		
			Recovery not specified		
			Protein organic solvent precipitation (PROSPR)		
			2 ml of plasma		
			Recovery not specified		

Since it was noticed that extracellular nanoparticles have clinical potential due to their cargoes, several discoveries occurred in this field. Recently, in addition to the classical EVs, two other nanovesicle groups lacking a lipid bilayer membrane have been reported: exomeres and supermeres. Special attention was given to the supermeres because they appear to be enriched in proteins and other molecules (Zhang et al., 2021).

Small extracellular vesicles (sEVs), which includes the well-known exosomes, are the most studied vesicle group, may be found in body fluids and cell-derived conditioned media, vary between 40 and 150 nm, and arise from the fusion of endosomal multi-vesicular bodies with the plasma membrane released by exocytosis by several cell types (Raposo and Stoorvogel, 2013; Ramirez et al., 2018). The internal composition of sEVs includes lipids; nucleic acids, such as messenger RNAs (mRNA), miRNA, and non-coding RNAs (ncRNA); and proteins (CD9, CD63, and CD81) (Théry et al., 2006; Colombo et al., 2014).

sEVs may be obtained from MSC conditioned culture media or body fluids (Table 1) for studies of their biogenesis, composition, and mechanisms of action. Ultracentrifugation is generally used for sEV concentration in a sucrose density gradient, whereas polymer precipitation is widely used to produce large quantities of EVs (Table 2). Assays that lead to co-precipitation of these vesicles with soluble proteins and supplemental chemical methods are also used to isolate EVs from conditioned media. Immunoaffinity methods and sEV extraction kits have also been employed to enable the isolation of these EVs in a shorter period of time (Raposo and Stoorvogel, 2013; Schageman et al., 2013; Rocco et al., 2016; Sarvar et al., 2016; Silva et al., 2016). Each method presents different advantages that should be evaluated according to application, prioritizing processing time, purity, cost, ease of handling process, or scalability. However, their structure and content should be preserved.

**TABLE 2:** Different kinds of MSCs used to isolate sEVs with different applications with details of the sEV isolation method and results of each work.

MSCs source of EVs	Application/purpose	Method of EVs isolation	Quantity of EVs obtained/applied	Effectiveness/results	References
Human Umbilical Cord MSCs (hucMSCs-exos)	The study explored the functional roles of uMSC exosomal microRNAs (miR-21/miR-23a/miR-125b/miR-145) in the process of skin wound healing, especially regarding myofibroblast formation	Ultracentrifugation	Yield obtained	Exosomal miRNAs studied seem to play roles in suppressing myofibroblast formation, which is closely involved with excessive scarring, by inhibiting *a*-smooth muscle actin and collagen deposition. Both processes are associated with the activation of the transforming growth factor-β/SMAD2 signaling	Fang et al. (2016)
			Not specified		
			Treatment		
			100 μg/ml uMSC-exo + 10 mg/ml hydrogel (ratio 1:1)		
3D-exosomes derived from human umbilical cord MSCs	Evaluated 3D culture system aiming to produce MSCs continuously and efficiently and to compare the efficacy of 3D MSCs-exos with conventional 2D culture in a murine model of cisplatin-induced acute kidney injury (AKI)	Ultracentrifugation + filtration	Yield obtained (protein)	Exosome production in 3D systems improved the efficiency of exosome collection. Also, 3D-exos show stronger renoprotection attenuating AKI conditions	Cao et al. (2020)
			2D—0.42 mg		
			3D—8.15 mg		
			Treatment		
			2 × 10^5^ incubated with 15 μg of exosomes (2D or 3D)		
Human umbilical cord MSCs (hucMSCs-exos)	Comprehend the role of hucMSCs -exos in wound healing using deep second-degree burn injury evaluating especially β-catenin signaling	Sucrose gradient + ultracentrifugation	Yield obtained not specified	Increase in wound closure by delivering Wnt4 to activate Wnt/b-catenin in skin cells and inhibition of acute heat stress-induced skin cell apoptosis *via* activation of AKT pathways increased the survival of skin cells	Zhang et al. (2015a)
			Treatment		
			200 µg (160 μg/ml)		
Human umbilical cord blood-MSC (HUCB-MSC)	Observe the expression and role of DMT1 in acute myocardial infarction (AMI) model exploring if HUCD-MSC exosomes inhibited ferroptosis *via* miR23a-3p/DMP1 axis to attenuate myocardial injury	Total exosome isolation (TEI)	Yield obtained not specified	Initially, it was shown that DMT1 was upregulated at 24 h after the establishment of an *in vivo* AMI model increasing cell ferroptosis. Also, HUCB-MSC exos suppress the ferroptosis of cardiomyocyte mediating myocardial repair in AMI mice *via* delivering miR-23a-3p	Song Y et al. (2021)
			Treatment		
			5 μg in 20 μl of PBS		
Human placenta (38 weeks) MSCs (Pla-MSC-exos)	Evaluate angiogenesis promotion by Exosomes *in vitro* and *in vivo*	Ultracentrifugation	Yield obtained 5.8 × 10^11^–7.6 × 10^11^ particles/10^6^ cells	Enhanced angiogenesis *in vitro* and in a murine auricle ischemia model, suggesting that exosomes play a role in the pro-angiogenic activity of Pla-MSCs	Komaki et al. (2017)
			Treatment		
			50 μl day		
Human exfoliated deciduous teeth MSCs (SHED-exos)	Explore the potential of stem cells from human SHED-exos for the management of traumatic brain injury (TBI) in rats	ExoQuick	Yield obtained not specified	Improvement in rat motor functional recovery and reduction of neuroinflammation after TBI by shifting microglia M1/M2 polarization	Li Y et al. (2017)
			Treatment		
			50, 100, 200, and 400 μg/ml		
Human adipose MSCs (ADSCs-exos)	Elucidate whether A-MSC-exos might induce neovascularization and protect skin flaps during ischemia/reperfusion (I/R) injury	ExoQuick-TC	Yield obtained (protein)	Significantly increased flap survival and capillary density, ADSC-exos increased angiogenesis and enhanced recovery from I/R injury in the long thoracic artery pattern of skin flaps in mice *via* IL-6 secretion	Pu et al. (2017)
			2.1 mg/ml (from 1 × 10^6^ cells)		
			Treatment		
			100 μg/ml		
Adipose tissue-derived MSCs	Evaluate whether MSC-derived exosomes could act as carriers of miR-199a-3p to enhance hepatocellular carcinoma (HCC) chemosensitivity	MagCapture exosome isolation kit (WAKO)	Yield obtained not specified	Apparently, miR-199a-3p-modified AMSC-exos can effectively increase the sensitivity of HCC cells to chemotherapeutic agents	Lou et al. (2020)
			Treatment		
			50 μg total protein in 200 μl of PBS		
Human synovial MSCs (SMSCs-exos)	Generate SMSCs-exos with miR-140-5p and evaluate their effectiveness in osteoarthritis (OA) therapy and prevention in the rat model	Ultrafiltration + sucrose gradient + ultracentrifugation	Yield obtained not specified	SMSC-140-exos were able to promote chondrocyte proliferation and migration with less influence on the secretion of extracellular matrix (ECM) and successfully prevented OA in a rat model	Tao et al. (2017b)
			Treatment (*in vitro*)		
			0, 1, 5, or 10 × 10^11^ particles/ml (*in vivo*) 100 μl (10^11^ particles/ml)		
Human induced pluripotent stem cell-MSCs (hiPSC-MSCs-exos)	To evaluate the therapeutic effects of hiPSC-MSCs-exos on hepatic ischemia-reperfusion (I/R) injury *in vitro* and in a murine model	Differential centrifugation + ExoQuick	Yield obtained not specified	hiPSC-MSCs-exos have hepatoprotective effects on hepatic I/R injury *via* activation of sphingosine kinase and sphingosine-1-phosphate pathway in hepatocytes, promoting cell proliferation	Du et al. (2017)
			Treatment (*in vitro*) 200 μg/ml (*in vivo*) 2.5 × 10^12^ particles in 500 μl PBS		
Rabbit bone marrow HIF-1α modified MSCs (BMSC-Exo^MU^)	Report the role of exosomes secreted by mutant HIF-1α-modified BMSCs in the recovery of the early Steroid-Induced Avascular Necrosis of Femoral Head (SANFH)	Total exosome isolation kit (TEI)	Yield obtained not specified	BMSC-Exos^MU^ facilitates the repair of SANFH by enhancing osteogenesis and angiogenesis in a rabbit model	Li Y et al. (2017)
			Treatment (*in vitro*) 10 μg/ml (*in vivo*) 20, 40 and 80 μg/ml protein in 0.5 ml PBS		
MiR-223-3p transfected rat bone marrow MSC-derived exosomes	Investigate exosomal miR-223–3p effect in cerebral inflammation through the modulation of CysLTR-mediated microglia polarization	Serial differential centrifugation + filtration	Yield obtained not specified	Exosomal miR-223–3p seems to be a candidate to attenuate cerebral inflammation during brain damage. This protective effect is highly associated with polarization of microglia phenotype	Zhao et al. (2020)
			Treatment (*in vitro*)		
			5 μg/ml (low)		
			10 μg/ml (medium)		
			20 μg/ml (high)		
Bone marrow MSCs educated with neonatal and adult mice serum-derived exosomes	Investigate and compare the effect of educated exosomes from neonatal and adult mice serum regarding their biological and therapeutic effect on cutaneous wound repair	Ultracentrifugation	Yield obtained 25 μl from 1 ml of serum	Exosomes from educated MSCs could promote angiogenesis by regulating biological properties of MSCs, such as their potential to differentiate and proliferate	Qiu et al. (2020)
			Treatment		
			100 μg of exosomes in 100 μl of PBS		
Atorvastatin (ATV) pretreated human bone marrow MSCs	Evaluate the potential of exosomes extracted from MSC pretreated with ATV to enhance biological properties of endothelial cells facilitating angiogenesis potential in cutaneous diabetic wounds and investigate the role of miRNAs in this context	Ultracentrifugation	Yield obtained Not specified	ATV treatment seems to enhance exosome function by promoting angiogenesis *via* upregulation of AKT/eNOS pathways through miR-221-3p, demonstrating pro-angiogenic effect	Yu et al. (2020)
			Treatment		
			50 μg/ml combined with high glucose (HG)		

Numerous techniques have been used to describe the morphology and evaluate the molecular content of sEVs. The focus has been to gather the largest amount of information about their biogenesis and mechanism of action. In addittion, protocols need to be standardized to illustrate their interaction with the target cells and their activity *in vitro* and *in vivo*. Among the most widely used techniques are transmission electron microscopy (TEM), proteomics, lipidomics, genomics, metabolomics, RNA sequencing, nanoparticle tracking analysis (NTA), immunological labeling, and classification by flow cytometry (Feng et al., 2010; Vallabhaneni et al., 2014; Anderson et al., 2016; Greening et al., 2016; Silva et al., 2016).

In pairwise proteomic and lipidomic comparative studies of EV from normal (bone marrow mesenchymal stem cells) and transformed cells (U87-MG human glioblastoma cells, Huh7 hepatocellular carcinoma cells) identified different expression profile of proteins and lipids between normal and tumor cells (Haraszti et al., 2016). Gene ontology terms related to vesicles and membrane-associated proteins, GTPases, translation, and glycolytic pathways were significantly enriched features of EVs contents. Concerning the lipid content, while sEV were rich in glycolipids and free fatty acids, medium EV displayed larger amounts of ceramides and sphingomyelins (Haraszti et al., 2016).

The specific set of molecules present on the surface and in the interior of the sEV, may enable cell targeting at various levels, mediating intercellular signaling and resulting in regulation of embryonic tissue pattern remodeling and homeostasis, as well as disease progression (Vyas and Dhawan, 2017). Therefore, the molecular composition of sEV has been widely studied, using numerous methodological approaches due to the concrete possibility of using them as diagnostic biomarkers for various diseases and as drug delivery agents and therapeutic RNAs (Lv et al., 2015; Deng and Miller, 2019). Also, studies have shown that MSC-derived EVs display similar composition and function compared to the cells they derive from, being able to exert therapeutic activity, thereby opening new avenues for cell-free therapy (Bollini et al., 2013; Zhang B et al., 2016). sEVs have been successfully employed as a cell-free therapy due to their small size, which facilitates their storage, with no embolus formation concerns, in addittion to being poorly or no immunogenic and reducing the concern of tumor formation upon intravenous application because no cells are involved (Rezakhani et al., 2021).

## Mechanisms of action of sEV

sEV have been responsible for the exchange of information among cells through various interaction mechanisms. Their bilipid membrane allows greater versatility of extracellular contact by receptor–ligand interactions (van Niel et al., 2018).

These interactions may occur through direct contact between proteins on the surface of the exosomes and signaling receptors on the target cell, as exemplified by the discovery of Purushothaman et al. (2016). Fibronectin on the surface of myosomal cell-derived exosomes facilitated the interaction of target cells in a mechanism where heparan sulfate proteoglycans are present in the exosomes and the target cells, appearing to play a dual role, allowing for fibronectin capture and reception.

A study showed that exosomes display on their surfaces support for binding and activation of bioactive proteins, as described in exosomes derived from human mast cells, which present the active and the latent forms of transforming growth factor-β1 (TGF-β1). TGF-β1 is transferred to MSCs and retained in endosomal compartments, being activated by the action of proteoglycans, heparinase-II, and pH-sensitive elements, resulting in prolonged signaling and migratory phenotype in the receptor cells (Shelke et al., 2019).

sEVs can also fuse with the target cell membrane or be endocytosed, allowing them to deliver all of their intra-vesicular content to the cell, acting as paracrine effectors (Toh et al., 2016). Upon endocytosis, sEVs are internalized by the target cells and then fused to endosomes, which can then undergo transcytosis, moving the sEV through the fused endosomes, where they may mature into lysosomes to be degraded (Mulcahy et al., 2014; Zhang et al., 2015). This mechanism was exemplified by Kita et al. (2019), with sEV from MSCs that migrate into ischemic areas and fuse themselves with neurons, thereby promoting neurogenesis and angiogenesis after stroke damage, resulting in relieving inflammation and lesions. Another example was reported by Feng et al. (2010) in which sEV from K562 human erythroleukemia cells and MT4 human T-lymphotropic virus type-1 (HTLV) transformed leukemia T cells could interact with different cells in two distinct manners: efficient internalization by phagocytic cells and by non-phagocytic cells. However, most sEVs remained anchored to the cell membrane, and the few intracellular sEVs were involved by cell extensions and large phagosomes and also co-localized with phagolysosome markers, indicating that they would be drawn into phagolysosomes.

MSC sEV can load and transfer their charge to parenchyma cells facilitating cerebral plasticity and functional recovery from stroke, acting as the main paracrine effectors, targeting specific modifications in miRNAs, aiming to modulation of therapeutic responses (Xin et al., 2014).

After administration, MSC-derived sEV from induced human pluripotent cells were able to fuse with target hepatocytes and increase the activity of the sphingosine kinase signaling pathway and the levels of sphingosine-1-phosphate, which is directly related to the protective and proliferative effects of these sEV. This mechanism may contribute to hepatic regeneration in a murine model of liver ischemia and reperfusion injury (Du et al., 2017).

Another widely used treatment approach is the application of platelet-rich plasma (PRP), which has been considered effective due to the presence of numerous growth factors and other bioactive molecules responsible for promoting tissue repair (Hara et al., 2016) in processes such as chronic wounds (Ostvar et al., 2015; Spanò et al., 2016) and burns (Marck et al., 2016). However, this technique is not officially standardized, and little is known about the mechanism(s) underlying PRP application (Pavlovic et al., 2016). In this sense, the discovery of platelet-derived sEV that can easily be obtained from the blood has raised great interest because they play key roles in angiogenic and proliferative processes in tissue regeneration, becoming good candidates to replace PRP or even being the “next-generation PRP” (Tao et al., 2017b) with low immunogenic potential. Platelet-derived EVs have also been chosen because they constitute most of the vesicles present in the bloodstream, allowing studies exploring their role in hemostasis, pro-coagulant activity, and their participation as biomarkers in pathologies associated with thromboembolic events, such as atherosclerosis (Goetzl et al., 2017; Tripisciano et al., 2017).

## MSC-derived sEV: Potential and efficacy for tissue regeneration

Several stimuli need to accompany tissue regeneration that is subdivided into three phases: inflammation, repair, and remodeling (Stroncek and Reichert, 2008). All these steps involve four molecular processes: a) attenuation of apoptosis, helping to prevent extensive cell loss; b) inflammation controlled by modulation of the immune system; c) migration of endothelial cells to promote angiogenesis; and d) repopulation of lost cell types through cell proliferation and differentiation. Generally, the injury process activates tissue cells, including epithelial, stromal, and resident immune cells, which induce the recruitment of other circulating immune cells, stimulating cytokine and growth factor secretion and initiating the inflammatory response. Then, phagocytosis and the removal of foreign and pathogenic bodies from the site of injury will occur. After the cleansing of dead and infected cells, the proliferative phase is reached. This phase is characterized by cell proliferation and migration to the lesion site, new deposition of the extracellular matrix, angiogenesis, formation of granular tissue, and re-epithelization. Subsequently, the remodeling phase starts, being characterized by extracellular matrix remodelling and scar maturation. The effectiveness of MSC-derived EVs has been reported at each one of these phases (Clark et al., 2007; Gurtner et al., 2008; Choi et al., 2012; Silva et al., 2016; Sorg et al., 2017; Wu et al., 2018). Also, utilization of the mesenchymal stem-cell secretome can positively influence the injured tissue by modulating the local microenvironment, providing cytoprotective, anti-inflammatory, and angiogenic effects during the acute regenerative phase, either by attenuating or by inducing the action of pro-inflammatory cytokines (Zhang et al., 2014), boosting resident progenitors/stem cells in place to achieve a more tissue-specific programmed repair and instructing the stimulation of resident endogenous progenitors (Bollini et al., 2013). In addition, cleansing enzymes are biochemically active in the proteome of MSC sEV associated with the restoration of homeostasis for the most important activities in the tissue environment, constituting an interesting mechanism to be targeted for future development of paracrine pharmacological therapy (Toh et al., 2016). Despite the need for further studies, the advantage of low or no immunogenicity displayed by MSC-derived sEV influences their use as drug delivery, therapeutic agents in vaccination, cancer biomarkers and in inflammatory disorders (Robbins and Morelli, 2014) of both the nervous (Lv et al., 2015) or cardiovascular system (Pankajakshan and Agrawal, 2014), and in wound healing (Geiger et al., 2015; Elahi et al., 2019). Considering that, 19 studies were found at the Clinicaltrials.gov (Table 3) website upon searching for “MSC+exosome” (https://clinicaltrials.gov/ct2/results?cond=&term=MSC+exosomes&cntry=&state=&city=&dist=), several focused on exploring their efficiency in tissue regeneration in diseases such as epidermolysis bullosa, acute ischemic stroke, and degenerative meniscal injury. Others aim to test the therapeutic effects of MSC-derived sEV in Alzheimer’s disease and COVID-19-associated pneumonia (Accessed on 20 July 2022, at 15:20 p.m.).

**TABLE 3:** Active clinical trials undertaken with mesenchymal stem cell-derived Extracellular vesicles in different disease treatments.

ID (clinical trials)	EVs (cells)	Purpose	Treatment	Phase status
NCT04798716	Mesenchymal stem cells (tissue not specified)	Treatment of novel coronavirus pneumonia and acute respiratory distress syndrome	Intravenously every other day on escalating dosage (2 × 10^9^ – 4 × 10^9^ – 8 × 10^9^)	Not yet recruiting—Phase 1
NCT05216562	Mesenchymal stem cells (tissue not specified)	Reduce hyper-inflammation in moderate COVID-19 patients	Intravenous injection on day 1 and day 7 of a 14-day study	Phase 2—Recruiting
			Obs: exosomes dissolved in 0.9% NaCl solution	
NCT03437759	Umbilical cord mesenchymal stem cells	Promote healing of large and refractory macular holes	After surgery, applying 50 μg or 20 μg of exosomes in 10 μl PBS dripped, directly, into the vitreous cavity	Active, not recruiting—Early Phase 1
NCT04356300	Umbilical cord mesenchymal stem cells	Treatment of multiple organ dysfunction syndrome after surgical repair for acute type aortic dissection	150 mg of exosomes will be injected intravenously once a day 14 times	Not yet recruiting
NCT04276987	Allogenic adipose mesenchymal stem cells	Severe Novel Coronavirus Pneumonia	2 × 10^8^ exosomes in 3 ml aerosol inhalation for 5 days consecutively	Completed
NCT05261360	Synovial fluid-derived mesenchymal stem cells	Evaluate the efficacy of exosomes in degenerative meniscal injury	Intra-articular administration—1 million cells/kg of exosomes	Recruiting—Phase 2
NCT05060107	Allogenic mesenchymal stem cells	Osteoarthritis (knee)	Intra-articular knee injection of 3–5 × 10^11^ particles/dose (single dose)	Not yet recruiting—Phase 1
NCT05402748	Human placenta mesenchymal stem cells	Treatment of complex anal fistula	Weekly injections for 3 consecutive weeks—dose not specified	Recruiting—Phase 1
NCT04388982	Allogenic adipose mesenchymal stem cells	Evaluated the efficacy of MSC-exos in Alzheimer’s disease patients	Nasal drip with 5, 10, and 20 μg (low, mid, and high doses, respectively) twice a week for 12 weeks	Recruiting—Phase 1
NCT04313647	Mesenchymal stem cells (tissue not specified)	Evaluated the tolerance of mesenchymal stem cell exosome aerosol inhalation (health volunteers)	2 × 10^8^ | 4 × 10^8^ | 8 × 10^8^ | 12 × 10^8^ | 16 × 10^8^ nanovesicles/3 ml	Completed
NCT03384433	Allogenic mesenchymal stem cells (tissue not specified)	Safety and efficacy of MSC-exosomes on patients with acute ischemic stroke	Exosomes enriched with miR-124 *via* stereotaxis/intraparenchymal 1 month after the attack	Recruiting
NCT04173650	Allogenic mesenchymal stem cells—product: AGLE-102	Safety and efficacy of MSC-exos in the treatment of lesions in patients with epidermolysis bullosa	Six administrations of MSC-exos in 3 months in a maximum of 50 cm^2^ of wound	Phases 1/2
NCT03608631	Mesenchymal stem cells (tissue not specified)	Study the best dose and side effect of MSC-exos in pancreatic cancer patients	Exosomes with KrasG12D siRNA—patients received on days 1, 4, and 10	Recruiting—Phase 1
NCT04602442	Mesenchymal stem cells (tissue not specified)	Safety and efficiency of exosomes inhalation in COVID-19 pneumonia	Inhalation of 3 ml of 0.5–2 × 10^10^/exosomes for 10 days	Enrolling by invitation—Phase 2

### Migration of endothelial cells to promote angiogenesis

A study with sEV derived from human placental MSCs pointed out that they can stimulate the transcriptional activity of Oct4 and Nanog in dermal fibroblasts. Then, by their incorporation, stem cells’ plasticity for differentiation into adipogenic and osteogenic lineages was considerably increased (Tooi et al., 2015). The pro-angiogenic capacity of sEV from the human placenta was also described *in vitro* using the ischemic lesion model of the murine atria, in which increased levels and migration of endothelial tubes were observed along with increased expression of genes related to angiogenesis (Komaki et al., 2017). Upon evaluating the therapeutic potential of human fibrocyte-derived sEV, a population of bone marrow-derived mesenchymal progenitors’ cells, sequentially stimulated with platelet-derived growth factor-BB (PDGF-BB) and TGF-β1 growth factors and in the presence of fibroblast growth factor (FGF), presented pro-angiogenic properties *in vitro*, induction of keratinocytes migration and proliferation, and an accelerated healing process in type 2 diabetic mice (Geiger et al., 2015).

Another interesting property of these vesicles is their ability to be internalized by human umbilical vein endothelial cells (HUVECs) *in vitro*, increasing their proliferation and migration, increasing their proloferation and migration accelerating the healing process of cutaneous wounds on the dorsal region of diabetic rats by stimulating angiogenesis through activation of the extracellular signal-regulated kinases 1/2 (Erk-1/2) signaling pathway, contributing to the discovery of specific pathways, which are activated during cutaneous regeneration (Zhang J et al., 2016).

### Repopulation of lost cell types through cell proliferation and differentiation

Acellular derivatives from bone marrow MSCs were described to accelerate wound closure by re-epithelialization, dermo-epidermal junction, regeneration of skin appendages, inflammatory infiltration, vascularization, and formation of granular tissue and higher density collagen fibers in diabetic mice, in addition to concentrate factors and proteins relevant to regeneration, compared to only bone marrow MSCs (de Mayo et al., 2017). Therefore, the ability to migrate to the site of injury and promote fibroblast migration and proliferation, together with collagen synthesis, has been described for human adipose tissue-derived MSC sEVs, contributing to the skin healing process in mice (Hu et al., 2016), as well as skin flap transplants through neovascularization and protection against ischemia/reperfusion, in addition to stimulating lesion recovery by increasing the interleukin- (IL-) 6 levels (Pu et al., 2017). Moreover, MSC-derived EVs from induced human pluripotent cells (iPSCs) could accelerate the healing process of cutaneous wounds in rats by stimulating the proliferation and migration of human fibroblasts motility, leading to increased collagen and elastin synthesis and stimulating blood capillary networks formation *in vitro* (Zhang et al., 2015b). This process could be regulated by different mechanisms. For example, fetal dermal MSC-derived EVs could promote cutaneous wound healing *in vivo* and *in vitro* by activating fibroblast motility and secretion ability *via* the activation of the Notch signaling pathway (Wang X et al., 2019). sEVs derived from synovial MSCs presented the potential to stimulate cell proliferation and migration and maintain the synthesis of extracellular matrix proteins, in addition to delaying the progression of osteoarthritis in an animal model of knee joint cartilage injury through miR-140-5p (Tao et al., 2017a).

### Attenuation of apoptosis helps prevent extensive cell loss

The wound healing process requires oxygen to promote cytokine interactions and activation of cell proliferation (Han and Ceilley, 2017). sEVs derived from bone marrow MSCs displayed rapid degradation of the hypoxia-inducible factor 1α gene (*HIF-1α*) under normoxic conditions. This effect is responsible for attenuating avascular necrosis of the steroid-induced femoral head in rabbits by promoting angiogenesis and accelerating bone regeneration, indicated by trabecular reconstruction and microvascular density (Li H. et al., 2017). The regeneration potential of EVs goes beyond wound repair. sEVs derived from human embryonic MSC demonstrated significant potential to reduce apoptotic rates and promote the proliferation of cells together with osteochondral differentiation (Zhang et al., 2018). Similar results were obtained using sEVs derived from human umbilical cord MSCs in an Alzheimer’s disease culture model. Results showed a high level of neuronal apoptosis *via* exosomal miR-223 targeting the PTEN-PI3K/Akt pathway, and this study demonstrated that EV derived from Alzheimer’s patients was capable of stimulating morphological changes, cell number reduction, and shortened synapses (Wei H et al., 2020).

Different studies have searched for a faster lung injury therapy aiming to relieve the idiopathic pulmonary fibrosis (IPF) condition, and during the COVID-19 pandemic, a race against time started aiming for alternative therapies for acute pneumonia from coronavirus infection. Thus, the inhalation of lung spheroid secretome derived from hypoxic sEV could attenuate the fibrosis scenario, decreasing collagen accumulation and myofibroblast proliferation (Dinh et al., 2020).

### Inflammation controlled by modulation of the immune system

In injury, resident skin cells are exposed to multiple danger signals known as damage-associated molecular patterns (DAMPs) and pathogen-associated molecular patterns (PAMPs) that will be recognized by the immune system, starting an inflammatory process (Landén et al., 2016). An interesting recently evaluated mechanism was that mouse adipose mesenchymal stem cells (ASCs) display the potential to polarize macrophages from the M1 to M2 phenotype *via* miR-21 secretion targeting the PI3K/Akt pathway, directly influencing the pro-angiogenic effects of these EVs (Zhu et al., 2020). Thus, sEVs derived from bone marrow MSCs induced macrophage M2 polarization *via* transfer of miR-223, accelerating the wound healing process (He et al., 2019). This mechanism was recently reported by Zhang et al., (2018), describing that MSC sEV treatment enhances the M2-macrophage infiltration and maintains this effect in cartilage. In contrast, M1 macrophage levels and inflammation-associated cytokines, such as IL-6 and tumor necrosis factor-α (TNF-α), were lower after EV treatment (Zhang et al., 2018). Such evidence highlight sEV microRNAs as a possible therapeutic target for tissue repair. In addition, human bone marrow MSC-derived sEVs could educate macrophages, resulting in an M2-like phenotype, which was then used to promote tendon healing in an Achilles tendon injury *in vivo* model *via* modulation of tissue repair and inflammation (Chamberlain et al., 2019). Additionally, accelerating wound healing without scar formation is the target of many studies aiming at specific components that sEVs could present. By analyzing sEVs from umbilical cord MSCs, specific miRNAs were observed, such as miR21, miR23a, miR125b, and miR145. Evidence has shown that they can play a key role in suppressing the formation of myofibroblasts and scars in *in vitro* and *in vivo* models by inhibiting the transforming growth factor-β2 (TGF-β2) and the SMAD2 pathway in mice (Fang et al., 2016). This suggests an alternative pathway to cell therapy by administering modified EVs with transfected miRNAs in wounds, preventing scar formation.

The most common COVID-19 symptom was lung injury produced by high levels of pro-inflammatory cytokines, such as TNF-α, granulocyte colony-stimulating factor (G-CSF), monocyte chemoattractant protein 1 (MCP-1), interferon- γ inducible protein 10 (IP10), IL-6, and IL-7 (Gupta et al., 2020; Jayaramayya et al., 2020). MSCs’ potential to stimulate the immune system by cytokine release and differentiation into other cell phenotypes called attention during the COVID-19 pandemic. Considering all that was previously discussed, human blood samples were analyzed, demonstrating elevated ACE2^+^ EV levels in COVID-19 patients with an even-higher rate in acute-inflammatory phase patients. Together, the infection inhibition potentials of ACE2^+^-EVs and ACE2^−^-EVs were compared, concluding that virus infection was blocked in the presence of ACE2^−^-EVs (El-Shennawy et al., 2022). Considering the variants tested, ACE2+EV can neutralize their infection, supporting their potential as an antiviral mechanism (El-Shennawy et al., 2022). Similar effects were demonstrated by comparing EV-ACE2 with soluble ACE (Cocozza et al., 2020).

Also, the potential of EVs was shown in neurological pathologies. Human exfoliated deciduous teeth (SHED) derived-sEVs were used as an alternative for the treatment of rat traumatic brain injury, reducing the neuro-inflammation resulting from the change in polarization of the microglia. In the end, sEV could improve motor function and reduce the cortical lesion in the *in vivo* test (Li Y. et al., 2017).

## Current challenges and future perspectives

This review highlights the high therapeutic potential of MSC-derived extracellular vesicles, which have been of growing interest as a promising tool for cell-free therapy. The standardization of extracellular vesicle isolation is already a reality with great improvements in recent years. However, the literature is very poor regarding fundamental information, such as the initial and final number of cells used to obtain EV, the exact content of EV and their respective protein amount. Moreover, most of the time, there is a lack of details about the methodology used for analysis. These drawbacks result in standardization issues and the impossibility of conducting comparative tests among different applications, hampering trials/ reprodutibility worldwide.

The action of these vesicles is demonstrated at all stages of the regenerative process, mediating cell migration and proliferation and stimulating angiogenesis in newly formed tissues until the recruitment of new cells. Despite that and their low immunogenic potential, compared to their host cells, further studies are needed to validate their efficacy and safety before employing them in clinical applications, as already occurring in different clinical trials shown previously. Issues such as the composition of EVs still need to be evaluated: what frequency should be used and the exact amount of EVs required in each application still require a rigorous investigation.

Moreover, considering their punctual action as an immunomodulatory molecule, the mechanism involved in EVs on tissue regeneration must be deeply investigated to avoid any adverse reaction that could harm the patients’ health after treatment. Taking this into account, a wide spectrum of questions is open to investigate and map which EV components act in each step of the regenerative process. The future perspective lies in strategically step into explore specific components of EVs cargo to modulate the body’s response for efficient tissue regeneration through target cell-free therapy.

## Conclusion

MSC EVs have shown great potential since they appear to have the same beneficial characteristics of cells application, such as the release of cytokines and growth factors. They are particularly used in tissue regeneration tests due to a vast number of reports in the last year uncovering a great deal of information on their biogenesis, isolation, and characterization processes. Currently, with the COVID-19 pandemic and the requirement for faster therapies, their application has been widely explored for coronavirus pneumonia and other diseases, such as tissue repair. This can be noted in the increasing number of clinical trials demonstrating that MSC-sEV present potential for tissue regeneration. Despite this progress, further studies in the field are needed to clarify misunderstood questions such as mechanisms of action and exactly which molecules participate in the regenerative process to provide a safe therapy approach.

## Abbreviations

MSCs, Mesenchymal/stromal stem cells; EVs, extracellular vesicles; mRNA, messenger RNA; miRNA, microRNA; ncRNA, non-coding RNA; TEM, transmission electron microscopy; NTA, nanoparticle tracking analysis; TGF-β1, transforming growth factor-β1; HTLV1, human T-lymphotropic virus type-1; TNF-α, tumor necrosis factor-α; IL-6, interleukin-6; IF-1α, hypoxia factor-1α; PDGF-BB, platelet-derived growth factor-BB; FGF, fibroblast growth factor; ASCs, adipose mesenchymal stem cells; TGF-β2, transforming growth factor; GCSF, granulocyte colony-stimulating factor; MCP1, monocyte chemoattractant protein-1; IP10, interferon- *γ* inducible protein 10; IL-7, interleukin-7; Erk-1, extracellular signal-regulated kinases-1; SHED, human exfoliated deciduous teeth; SHED-Exo, human exfoliated deciduous teeth-derived exosomes; PRP, platelet-rich plasma; P-exos, platelet-derived exosomes; sEVs, small extracellular vesicles.
